# Platelet Mitochondrial Function and Endogenous Coenzyme Q_10_ Levels Could Be Used as Markers of Mitochondrial Health in Infertile Men: A Pilot Study

**DOI:** 10.3390/ijms26010268

**Published:** 2024-12-31

**Authors:** Zuzana Sumbalová, Zuzana Rausová, Jarmila Kucharská, Patrik Šranko, Peter Harbulák, Pavel Svitok, Guillermo López-Lluch, Anna Gvozdjáková

**Affiliations:** 1 Institute of Medical Chemistry, Biochemistry and Clinical Biochemistry, Faculty of Medicine, Comenius University Bratislava, Sasinkova 2, 811 08 Bratislava, Slovakia; 2 Pharmacobiochemical Laboratory of 3rd Department of Internal Medicine, Faculty of Medicine, Comenius University Bratislava, Sasinkova 4, 811 08 Bratislava, Slovakia; zuzana.rausova@fmed.uniba.sk (Z.R.); jarmila.kucharska@fmed.uniba.sk (J.K.); 3 GYN-FIV, a.s., Trnavská cesta 106, 821 01 Bratislava, Slovakia; patrik.sranko@gyn-fiv.sk (P.Š.); peter.harbulak@gyn-fiv.sk (P.H.); pavel.svitok@gyn-fiv.sk (P.S.); 4 Department of Physiology, Anatomy and Cellular Biology, Pablo de Olavide University, 41013 Seville, Spain; glopllu@upo.es; 5 Faculty of Medicine, Slovak Medical University, Limbová 12, 833 03 Bratislava, Slovakia; anna.gvozdjakova@fmed.uniba.sk

**Keywords:** mitochondria, platelets, bioenergetics, oxidative stress, coenzyme Q_10_, tocopherols, infertility, oxidation–reduction potential, semen quality, spermatozoa

## Abstract

Fertility disorders are a worldwide problem affecting 8–12% of the population, with the male factor substantially contributing to about 40–50% of all infertility cases. Mitochondria, crucial organelles for cellular viability, play a pivotal role in the processes of spermatogenesis and significantly affect sperm quality and their fertilizing ability. Mitochondrial oxidative phosphorylation (OXPHOS) dysfunction, reduced energy supply for sperm, reduced endogenous coenzyme Q_10_ (CoQ_10_) levels, and oxidative stress are among the main factors that contribute to male infertility. There is great interest in the role of mitochondrial dysfunction in male infertility, and the diagnosis and assessment of mitochondrial health in infertile men present challenges. Platelets are a source of viable mitochondria that can be obtained non-invasively. Changes in platelet mitochondrial respiration were documented in various diseases, confirming platelet mitochondrial bioenergetics as a marker of systemic mitochondrial health. The aim of our study was to determine whether (a) platelet mitochondrial bioenergetics and CoQ_10_ levels could be used as metabolic markers of mitochondrial health in infertile men and whether (b) the parameters of mitochondrial respiration in platelets correlate with sperm parameters. The high-resolution respirometry method was used for platelet bioenergetics, and the high-performance liquid chromatography (HPLC) method was used for CoQ_10_ level measurement. The static oxidation–reduction potential (sORP) of the ejaculate was evaluated by MiOXSYS^®^System. We found a deficit in mitochondrial complex I-linked OXPHOS and electron transfer capacity and CoQ_10_ and α-tocopherol levels in infertile men. The proportion of sperm, heads, and midpiece abnormalities correlated negatively with the complex I-linked parameters of platelet mitochondrial bioenergetics. We suppose that dysfunctional mitochondria contribute to increased oxidative stress, and these imbalances can be considered a cause of Male Oxidative Stress Infertility (MOSI). Our results suggest that platelet mitochondrial function and the endogenous levels of CoQ_10_ in platelets could be used as metabolic markers for monitoring mitochondrial health and targeted therapy in infertile men. sORP could be a useful clinical biomarker of MOSI.

## 1. Introduction

Reduced sperm quality, abnormal sperm morphology, impaired sperm mitochondrial function, deoxyribonucleic acid (DNA) fragmentation, and oxidative stress contribute to reduced fertility in men, decreasing the natural pregnancy rate in women. Infertility is defined as failure to conceive after 12 months of unprotected sexual intercourse [1]. According to the WHO, this worldwide problem affects 8–12% of the population, and males contribute to approximately 40–50% of the overall cases [2]. There are generally three types of risk factors contributing to infertility: congenital, acquired, and idiopathic. Male fertility tends to decrease with age and is negatively affected by obesity, alcohol intake, smoking, stress, environmental pollutants, and chemicals [3,4]. New molecular causes of infertility are being identified, and novel laboratory methods are being introduced. The areas of metabolomics, proteomics, transcriptomics, genomics and epigenomics (gene expression and DNA methylation), DNA integrity and structure (TUNEL, Comet, etc.), and oxidative stress markers (sORP) have become the target of modern diagnostics [5]. Impaired male fertility is often associated with a genetic background where DNA mutations, Y chromosome microdeletions, telomere elongation, centromere aberrations [6], and changes in mitochondrial DNA (mtDNA) are observed. Although mitochondria are inherited exclusively from the mother, mutations and deletions of mtDNA genes are associated with defects in important sperm functions and successful fertilization [7].

Male infertility is associated with decreased sperm mitochondrial function. Mitochondria play an important role in cellular energy metabolism; they are the main producers of energy in the form of adenosine triphosphate (ATP). In the male reproductive system, mitochondria are important for the development of healthy sperm. Mature sperm is composed of a head with a nucleus, a midpiece with mitochondria, and a tail. The midpiece contains 72–80 mitochondria helically wrapped around the axoneme, forming the mitochondrial sheath with 10–12 mitochondrial gyres. In the midpiece, ATP essential for promoting sperm motility is produced by mitochondria through oxidative phosphorylation (OXPHOS) [8]. Any alternation in the quality of sperm mitochondria may cause male infertility [9]. Mitochondrial anomalies that are related to midpiece defects can include the following: irregular organization, abnormally shorter or longer mitochondrial sheaths, increased matrix density, lipid inclusions, localized or total absence of mitochondria from the midpiece, lack of the midpiece, or the local aggregation of mitochondria with normal ultrastructures [10]. Abnormally assembled mitochondria, small midpieces, and mitochondrial membranes with structural defects have been associated with low sperm motility [8]. Coenzyme Q_10_ (CoQ_10_) is a key component of the mitochondrial respiratory system that is necessary in the process of ATP production. It serves as a mobile carrier in the inner mitochondrial membrane, accepting electrons from complex I (CI), complex II (CII), and other electron-transferring flavoproteins including the glycerophosphate dehydrogenase complex (CGpDH). From CoQ_10_, electrons flow linearly through complex III, cytochrome *c*, and complex IV to oxygen [11]. A deficit of CoQ_10_ negatively affects electron transfer and the phosphorylation of adenosine diphosphate (ADP) in the mitochondrial respiratory system. CoQ_10_ is present in all intracellular membranes and is one of the most important lipid-soluble antioxidants that protect membranes against oxidative damage [12,13]. The significant impacts of CoQ_10_ on sperm count and motility have been documented [14,15].

The aim of our study was to determine whether platelet mitochondrial bioenergetics, a marker of systemic mitochondrial health, and the endogenous levels of CoQ_10_ in platelets—a key component of the mitochondrial respiratory system for ATP production—could be used as markers of mitochondrial health in men with impaired fertility. Additionally, we wanted to find out whether the parameters of mitochondrial bioenergetics in platelets correlate with the parameters of sperm function or abnormal sperm morphology.

## 2. Results

### 2.1. Semen Parameters in Infertile Men

Conventional semen analysis was performed for all infertile couples. The mean values ± sem of semen parameters and the number and proportion of patients with normal values are given in Table 1.

All patients had pH values above the fifth percentile. Ejaculate volume was above the fifth percentile in 93.3% of the patients, total sperm number was above the fifth percentile in 80.0% of the patients, and sperm concentration and proportion of motile sperm were above the fifth percentile in 76.7% and 70% of the patients, respectively. In contrast, progressive motility above the fifth percentile was only in 23.3% of the patients, and the normal proportion of morphologically healthy sperm above the fifth percentile was observed in only 26.7% of the patients. Oxidation–reduction potential measurements showed normal values (0–1.38 mV/10^6^ sperm/mL) in the ejaculate of 50% of the patients. Based on the manufacturer’s data, values above 1.38 mV/10^6^ sperm/mL reflect high oxidative stress, and values above 1.41 mV/10^6^ sperm/mL are associated with reduced fertilization potential of sperm [16]. sORP in the ejaculate of 50% of the patients was above 1.41 mV/10^6^ sperm/mL, predicting low fertilization potential of their sperm.

### 2.2. Platelet Mitochondrial Respiration and OXPHOS in Infertile Men

**Figure 1:**
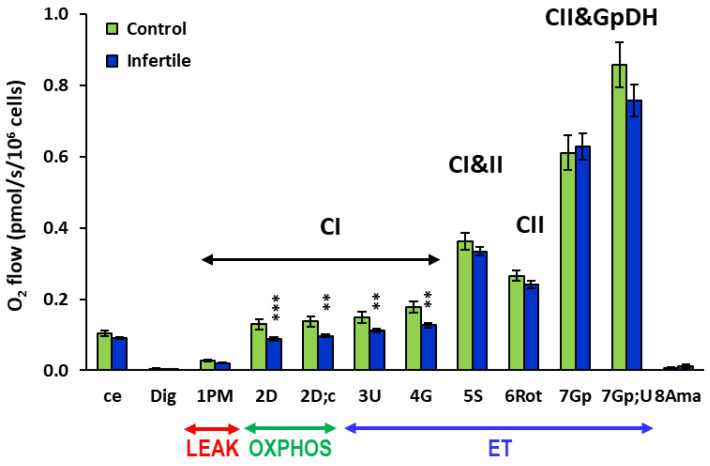
Platelet mitochondrial respiration and ATP production in infertile men. Parameters of platelet mitochondrial respiration and ATP production in healthy controls and infertile men, expressed as O_2_ flow (pmol/s/10^6^ cells). The evaluated respiratory capacities are marked according to the titration steps in the substrate–uncoupler–inhibitor titration (SUIT) reference protocol 1 [17] and correspond to the following respiratory states: ce—routine respiration of intact cells; Dig—residual oxygen consumption (ROX) after permeabilization with digitonin; 1PM—LEAK respiration with CI-linked substrates pyruvate + malate; 2D—CI-linked OXPHOS capacity (associated with ATP production); 2D;c—CI-linked OXPHOS capacity after the addition of cytochrome *c* as a test for the integrity of the outer mitochondrial membrane; 3U—CI-linked electron transfer (ET) capacity with pyruvate + malate; 4G—CI-linked ET capacity with pyruvate + malate + glutamate; 5S—CI&II-linked ET capacity after the addition of succinate; 6Rot—CII-linked ET capacity after the inhibition of CI by rotenone; 7Gp—CII&GpDH-linked ET capacity after the addition of glycerophosphate; 7Gp;U—real CII&GpDH-linked ET capacity after the additional titration of an uncoupler; 8Ama—ROX after the inhibition of mitochondrial CIII by antimycin A. CI—respiration related to mitochondrial CI activity, CI&II—respiration related to mitochondrial CI and CII activity, CII—respiration related to mitochondrial CII activity, CII&GpDH—respiration related to mitochondrial CII and glycerophosphate dehydrogenase (GpDH) activity. All substrates were titrated in kinetically saturating concentrations, and the uncoupler FCCP was titrated in the optimum concentration to reach the maximum flux. LEAK—non-phosphorylating state of respiration, OXPHOS—the capacity of oxidative phosphorylation, ET—the capacity of electron transfer. Control—the control group (*N* = 16), Infertile—the group of infertile men (*N* = 30), ** *p* < 0.01, *** *p* < 0.001—statistically significant difference vs. the control group. For abbreviations and the protocol, see the Methods Section.

High-resolution respirometry revealed no significant difference in routine respiration of intact platelets (labeled ce) and CI-linked LEAK respiration (mitochondrial respiration of permeabilized platelets after the addition of digitonin and CI-linked substrates pyruvate and malate, labeled 1PM) between the group of patients and the control group. However, the parameters of platelet mitochondrial respiration representing CI-linked OXPHOS and electron transfer (ET) capacity were statistically significantly reduced in the infertile patients in comparison with the healthy subjects: the platelet mitochondrial respiration after the addition of ADP (CI-linked OXPHOS capacity labeled 2D and 2D;c) was reduced to 68.4% (*p* = 0.0009) and 70.5% (*p* = 0.0015), the respiration after the titration of an uncoupler (CI-linked ET capacity labeled 3U) was reduced to 75.1% (*p* = 0.006), and the respiration after the addition of glutamate (labeled 4G) was reduced to 71.7% (*p* = 0.0011) in the infertile patients compared to the healthy subjects (Figure 1). 

There was no statistically significant difference between the group of infertile patients and the control group in platelet mitochondrial respiration after the addition of succinate (the respiration labeled 5S), rotenone (labeled 6Rot), glycerophosphate (labeled 7Gp and 7Gp;U), and antimycin A (labeled 8Ama) (Figure 1).

### 2.3. Antioxidants in Blood, Plasma, and Platelets

**Figure 2:**
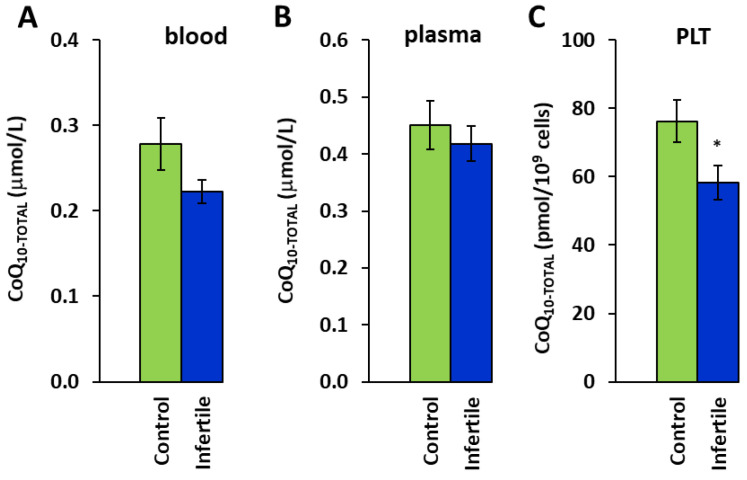
Coenzyme Q_10-TOTAL_ in blood (**A**), plasma (**B**), and platelets (**C**). Control—the control group, Infertile—the group of infertile men, * *p* < 0.05—statistically significant difference vs. the control group.

**Figure 3:**
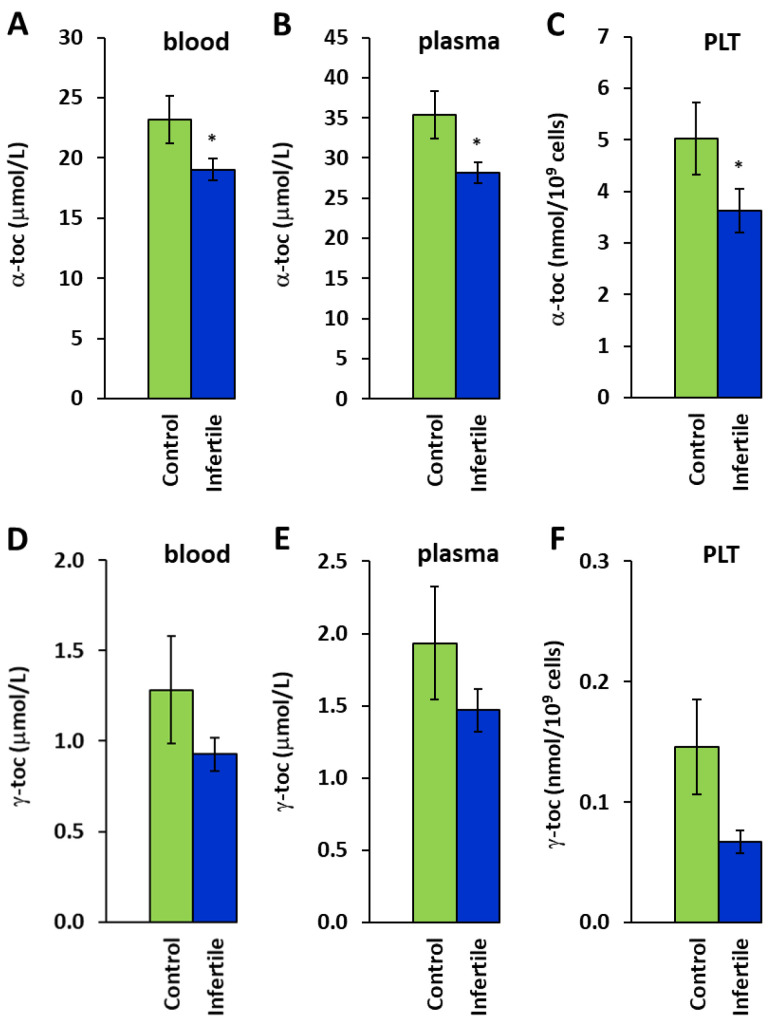
α-tocopherol in blood (**A**), plasma (**B**), and platelets (**C**), and γ-tocopherol in blood (**D**), plasma (**E**), and platelets (**F**). Control—the control group. Infertile—the group of infertile men. * *p* < 0.05—statistically significant difference vs. the control group.

The group of infertile men had lower concentrations of coenzyme Q_10_ and α- and γ-tocopherols (Figure 2 and Figure 3) compared to the healthy subjects.

The CoQ_10_ concentration was reduced to 76.5% (*p* = 0.034) in platelets (PLT) (Figure 2C). The reduction in CoQ_10_ concentrations in blood did not reach statistical significance. α-tocopherol concentrations were reduced to 82.1% (*p* = 0.043), 79.6% (*p* = 0.022), and 72.0% (*p* = 0.037) in blood, plasma, and PLT in comparison to the healthy men (Figure 3A–C). γ-tocopherol concentrations in the blood, plasma, and platelets of the infertile patients were also lower than those in the healthy controls; however, these declines did not reach statistical significance (Figure 3D–F).

### 2.4. Lipid Peroxidation in Plasma

Plasma thiobarbituric acid reactive substances (TBARS) levels in the group of infertile patients did not differ from the control group values, accounting for 4.98 ± 0.17 μmol/L in the control group and 4.18 ± 0.18 μmol/L in the group of infertile men.

### 2.5. Correlations

#### 2.5.1. Correlations Between Mitochondrial Respiration and CoQ_10_ Level in Platelets

**Figure 4:**
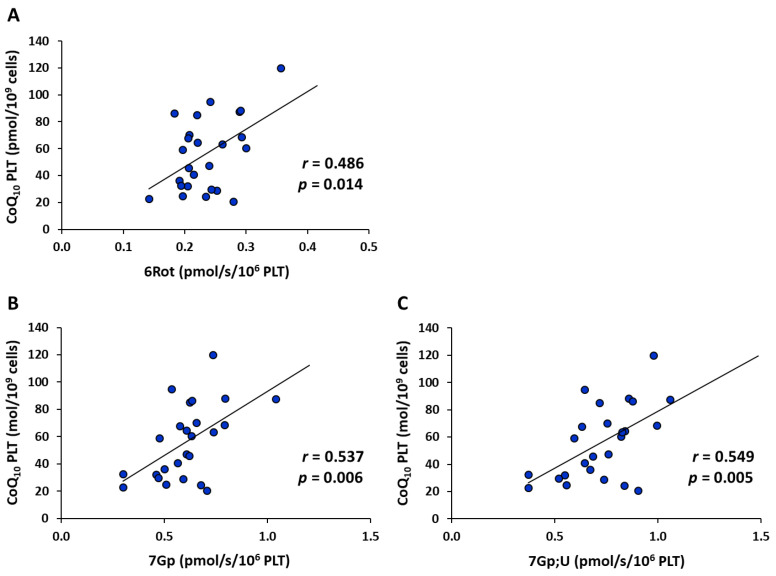
Correlations between parameters of mitochondrial respiration and CoQ_10_ concentration in platelets. Note: 6Rot (**A**)—the parameter of mitochondrial respiration representing CII-linked capacity of electron transfer; 7Gp (**B**), 7Gp;U (**C**)—the parameters of mitochondrial respiration representing CII&GpDH-linked capacity of electron transfer at suboptimal and optimal concentrations of an uncoupler; *r*—Pearson correlation coefficient; the *p*-value shows the statistical significance of the correlation.

Among all the parameters of mitochondrial respiration in platelets, only parameters 6Rot, 7Gp, and 7Gp;U correlated positively with the concentration of CoQ_10_ in platelets (*r* = 0.486, *p* = 0.014 for 6Rot, *r* = 0.537, *p* = 0.006 for 7Gp, and *r* = 0.549, *p* = 0.005 for 7Gp;U) (Figure 4). These results show the great importance of CoQ_10_ concentration in platelets for CII- and CII&GpDH-linked capacity of electron transfer. A deficit of CoQ_10_ may negatively affect these parameters, which means the electron flow from CII and CGpDH to oxygen may be limited by a low concentration of CoQ_10_.

#### 2.5.2. Correlations Between Mitochondrial Respiration and Various Sperm Abnormalities

**Figure 5:**
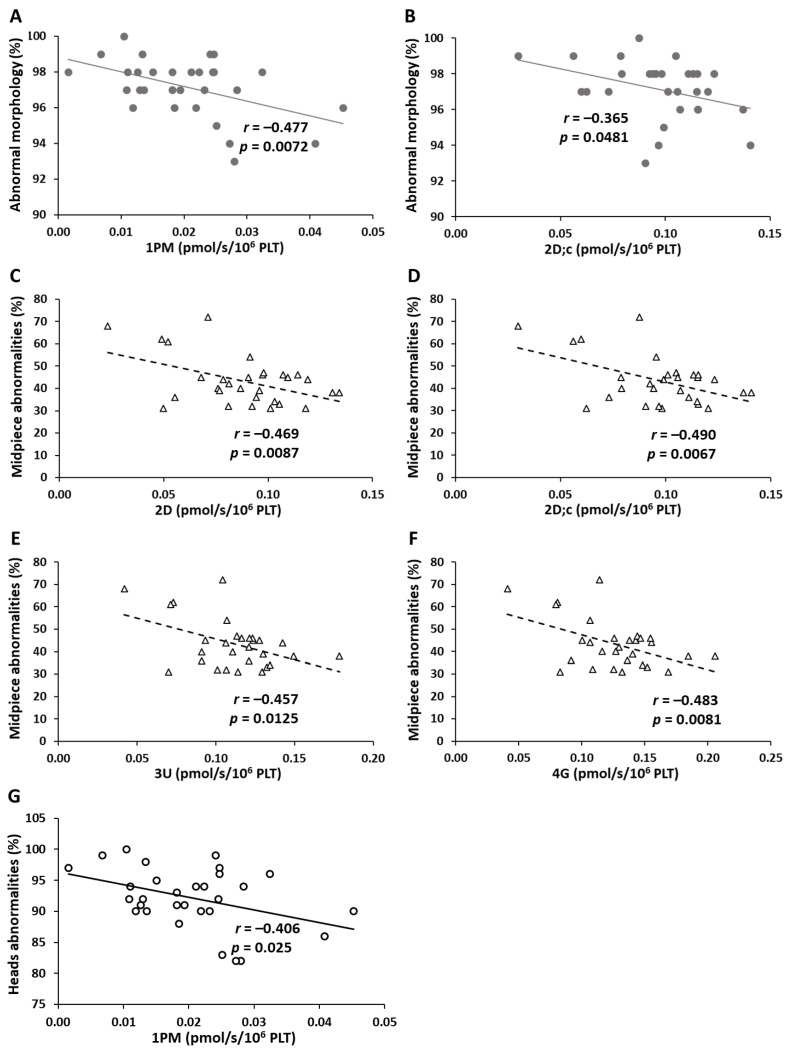
Correlation between the parameters of platelet mitochondrial respiration and ATP production and the proportion of abnormal sperm morphology (full circles, (**A**,**B**)), midpiece defects (triangles, (**C**–**F**)), and sperm head abnormalities (empty circles, (**G**)). Note: 1PM—CI-linked LEAK respiration with pyruvate + malate; 2D, 2D;c—CI-linked mitochondrial respiration associated with ATP production; 3U, 4G—CI-linked parameters of mitochondrial respiration representing ET capacity; *r*—Pearson correlation coefficient; the *p*-value shows the statistical significance of the correlation.

The analysis revealed significant correlations between the parameters of mitochondrial respiration in platelets and various sperm abnormalities. The significant correlations are shown in Figure 5. There were significant negative correlations between the proportion of abnormal sperm morphology and CI-linked LEAK respiration (1PM, *r* = −0.477, *p* = 0.0072) and CI-linked OXPHOS capacity (2D;c, *r* = −0.365, *p* = 0.0481) (Figure 5A,B). The proportion of midpiece defects negatively correlated with parameters representing CI-linked OXPHOS capacity (2D, *r* = −0.469, *p* = 0.0087 and 2D;c, *r* = −490, *p* = 0.0062) (Figure 5C,D), and CI-linked ET capacity (3U, *r* = −0.457, *p* = 0.0125 and 4G, *r* = −0.483, *p* = 0.0081) (Figure 5E,F). A significant negative correlation was also observed between the proportion of sperm head abnormalities and CI-linked LEAK respiration (1PM, *r* = −0.406, *p* = 0.025) (Figure 5G).

#### 2.5.3. Correlations Between Various Semen Parameters

There was an interdependence between various parameters of standard semen analysis and correlations between the parameters of standard semen analysis and sORP as a complex measure of oxidative stress in semen. The correlation coefficients *r* and the statistical significance (*p*-value) of the correlations are shown in Table 2.

The total sperm number correlated positively with the sperm concentration, the motile sperm concentration, and the proportion of sperm with progressive motility. The sperm concentration correlated positively with the concentration of motile sperm and with the proportion of sperm with progressive motility. The proportion of abnormal sperm morphology correlated negatively with the sperm concentration, the motile sperm concentration, and the proportion of sperm with progressive motility. The proportion of tail abnormalities correlated negatively with the total sperm number. The proportion of head and tail abnormalities correlated negatively with the sperm concentration, the concentration of motile sperm, and the proportion of sperm with progressive motility. The proportion of abnormal sperm morphology, the proportion of head abnormalities, the proportion of midpiece abnormalities, and the proportion of tail abnormalities positively correlated with each other (Table 2). The oxidation–reduction potential sORP correlated negatively with the total sperm number, the sperm concentration, the concentration of motile sperm, and the proportion of sperm with progressive motility. sORP positively correlated with the proportion of tail abnormalities (Table 2).

## 3. Discussion

Spermatogenesis is a complicated process of development of mature spermatozoa from germ cells in the seminiferous tubules of the testis. Developing sperm cells are nourished through the stages of spermatogenesis by Sertoli cells [18]. Spermatogenesis is highly dependent on optimal conditions for the process to proceed correctly. The formation of mature spermatozoa that are able to fertilize oocytes is essential for sexual reproduction. Ejaculate analysis provides basic information about the functional state of the male reproductive organs. The sixth edition of the WHO manual for the examination and processing of human semen shows the data from men in couples who achieved a pregnancy within one year of unprotected sexual intercourse leading to a natural conception. There are no clear thresholds in semen examination results and sperm defect indices between male partners of fertile and infertile couples; however, reduced sperm quality may contribute to the infertility of the couple [1].

Mitochondria play an important role in male and female fertility [4,19,20]. The motility of sperm requires a lot of energy and depends strongly on ATP produced by oxidative phosphorylation in sperm mitochondria. Compromised function of the mitochondria spirally arranged around the central axis of the axoneme can affect sperm motility and fertilization ability. Mitochondrial dysfunction leads to ATP depletion and is associated with excessive release of reactive oxygen species (ROS) from the mitochondrial respiratory chain, contributing significantly to oxidative stress that could lead to damage of proteins, lipids, and DNA including mtDNA. On the other hand, changes in mtDNA may have an impact on mitochondrial function and, consequently, on sperm motility [21]. Several mechanisms are involved in male infertility. Impaired mitochondrial OXPHOS in Sertoli cells causes disorders in energy metabolism and excessive ROS production, which leads to depolarization of the mitochondrial inner membrane, apoptosis, and ferroptosis [22]. While a low level of ROS is involved in sperm physiological processes [23], a high level of ROS and lipid peroxidation can lead to the loss of sperm motility and viability. Excessive ROS production is derived mainly from complexes I and II of the mitochondrial respiratory chain [24].

In our study, we evaluated mitochondrial function by high-resolution respirometry, using a comprehensive substrate–uncoupler–inhibitor titration protocol [17] to determine several mitochondrial pathways in one measurement. We determined the electron flow through CI, CII, and the glycerophosphate dehydrogenase complex (CGpDH). Mitochondrial glycerophosphate dehydrogenase (mGpDH) is the key enzyme connecting OXPHOS, glycolysis, and fatty acid metabolism. According to our results, in infertile men, only the CI-linked pathway was negatively affected. CI-linked platelet mitochondrial respiration and ATP production was decreased in the group of infertile men vs. the healthy control group. CI-linked OXPHOS capacity (the parameters 2D and 2D;c) was decreased to 68.4% and 70.5%, and CI-linked ET capacity (the parameters 3U and 4G) was decreased to 75.1% and 71.7% in comparison with the healthy subjects. The decrease in OXPHOS and ET capacity linked to complex I of the respiratory chain reflects the decreased activity of CI. The changes in the activity of CI negatively affect the amount of ATP produced by mitochondria and can significantly increase the formation of ROS by the mitochondrial respiratory chain, thereby increasing oxidative stress. Male Oxidative Stress Infertility (MOSI) is a condition in which oxidative stress (OS) contributes to abnormal semen characteristics, leading to infertility. OS can negatively affect fertility via a number of pathways, including interference with capacitation and possible damage to the sperm membrane and DNA, which may impair the sperm’s potential to fertilize an egg and develop into a healthy embryo [25].

Asthenozoospermia, defined as less than 42% of motile sperm in a semen sample, or less than 30% of sperm with forward motility, is a common cause of male infertility [26]. According to the WHO fifth edition, forward motility is a more important parameter of asthenozoospermia, and in 76.7% of the patients involved in our study, this parameter was below 30%. Another severe cause of male infertility is teratozoospermia, the condition when abnormal sperm morphology reaches over 96%. Teratozoospermia was observed in 73.4% of the patients involved in our study. Eighteen patients had a combination of both asthenozoospermia and teratozoospermia. Seven patients with oligozoospermia, the condition defined as a concentration of spermatozoa less than 16 × 10^6^/mL, had both asthenozoospermia and teratozoospermia. When the patients with asthenozoospermia (*N* = 23) were evaluated, the parameters of mitochondrial respiration in platelets were lower than in the group of all infertile patients: CI-linked OXPHOS capacity was decreased to 65.9% and 68.3% (the parameters 2D and 2D;c) and CI-linked ET capacity (the parameters 3U and 4G) was decreased to 72.7% and 69.3% in comparison with the healthy subjects. These parameters were even lower in the patients with teratozoospermia (*N* = 22), accounting for 64.0%, 66.2%, 71.0%, and 67.5%, respectively, of the control group values. These results point to the association between impaired mitochondrial function, defective sperm movement, and abnormal morphology. 

Our results are in accordance with the study by Cedíková et al. [27], showing decreased capacity of CI-linked OXPHOS in the sperm of men with asthenozoospermia. Decreased sperm count and motility, an increased proportion of midpiece defects, decreased ADP-stimulated respiration of sperm mitochondria, and increased parameters of oxidative stress in sperm were observed in patients with varicocele, being the most common cause of male infertility [28]. In our study, no patients were diagnosed with varicocele. We studied mitochondrial bioenergetics in platelets, which can reflect the mitochondrial health of the donor. The decreased capacity of platelet mitochondrial respiration and ATP production associated with CI could be a sign of oxidative stress.

We sought correlations between sperm abnormalities and parameters of mitochondrial bioenergetics in platelets and found a negative correlation between the proportion of midpiece defects and CI-linked OXPHOS and ET capacity. The proportion of abnormal sperm morphology and head abnormalities negatively correlated with CI-linked LEAK respiration. These results show that the defective function of mitochondrial CI can negatively affect the macroscopic structure of the sperm.

Coenzyme Q_10_ is a key component of the mitochondrial respiratory chain and an important antioxidant present in all types of membranes, protecting them against oxidative damage. In sperm, CoQ_10_ is concentrated in the mitochondria of the midpiece. The energy processes of sperm and its motility depend on the availability of CoQ_10_ [29]. It has been shown that the content of CoQ_10_ in seminal plasma directly correlates with seminal parameters such as sperm count, motility, and viability [14,30,31]. According to our results, the CoQ_10_ concentration was significantly lower in the platelets of the infertile men, representing 76.5% (*p* = 0.034) of the control group value. In the patients with asthenozoospermia, the concentration of CoQ_10_ in platelets represented 73.0% (*p* = 0.012), and in the patients with teratozoospermia, it represented 75.1% (*p* = 0.024) of the control group value. We suppose that low endogenous levels of CoQ_10_ might be the cause of aggravated seminal parameters.

The positive correlations between parameters of mitochondrial respiration in platelets associated with CII, CII&GpDH, and CoQ_10_ content in platelets suggest a negative effect of a CoQ_10_ deficit on mitochondrial respiration and ATP production. Previous studies showed that CoQ_10_ could be effective for infertility treatment: supplementation with CoQ_10_ increased sperm concentration and total and progressive sperm motility in infertile men [32,33,34]. CoQ_10_ administered together with vitamins and other nutritional supplements increased sperm motility and sperm concentration [35], increased sperm density, and decreased parameters of oxidative stress [36] in men with impaired fertility.

Antioxidants and trace elements are important for sperm development and quality; the most important are considered vitamin C, vitamin E, CoQ_10_, zinc, and selenium. Zinc is important for testicular development and sperm maturation, and selenium is important for testosterone biosynthesis and sperm formation. Sperm are protected from ROS-induced oxidative damage by antioxidants in seminal plasma. Decreased antioxidant activity may play a crucial role in the pathology of male infertility. The deficiency in antioxidants and trace elements may lead to increased OS, DNA damage, abnormal sperm morphology, and the loss of sperm motility and viability. The ROS concentration has been reported to inversely correlate with semen volume, the proportion of motile spermatozoa, and the linear movement of sperm in semen [15].

Vitamin E is a potent antioxidant involved in infertility [37]. Vitamin E is considered important for complete normal spermatogenesis in men, and its deficiency has been associated with reduced quantitative and qualitative spermatogenesis [38]. In a placebo-controlled clinical trial, supplementation with vitamin E prevented sperm from oxidative damage by neutralizing free radical activity [39]. Vitamin E is a group of eight fat-soluble compounds (four tocopherols and four tocotrienols) functioning as antioxidants protecting cell membranes from ROS. α-tocopherol is the form of vitamin E that is preferentially absorbed and accumulated in humans; the concentrations of γ-tocopherol in plasma and tissues are significantly lower [40]. The metabolism of γ-tocopherol is faster than that of α-tocopherol and takes place in the liver using a cytochrome P450-dependent process. γ-tocopherol acts as a fat-soluble antioxidant and nitric oxide (NO) radical scavenger, and due to NOx scavenging, it has potentially better protective effects on mitochondrial function than α-tocopherol [41]. Our results show significantly decreased levels of α-tocopherol in blood, plasma, and platelets of infertile patients vs. healthy men. An appropriate amount of vitamin E can effectively ameliorate sperm DNA damage in older men [42]. Supplementation with vitamin E significantly improved sperm parameters and increased the pregnancy rate, total sperm number, concentration, and progressive motility of the sperm [37]. The study by Keshtgar et al. [43] showed the in vitro protective effect of α-tocopherol on the motility and viability of teratozoospermic semen samples. Supplementation with γ-tocopherol has been shown to be beneficial in managing inflammation-associated diseases in animal models and some human studies [41].

In our study, we determined indicators of oxidative stress: the concentration of TBARS assessing lipid peroxidation in plasma and sORP—an integrated measure of the balance between the total levels of oxidants and antioxidants in the semen of infertile men. According to our results, there was no significant difference in the level of TBARS in the plasma of infertile patients vs. the healthy controls. On the other hand, sORP showed increased oxidative stress in the ejaculate of 50% of our patients, predicting the low fertilization potential of their sperm. sORP is considered the most complete measure of oxidative stress, surpassing existing tests focused on individual oxidative stress markers [44]. In our study, sORP negatively correlated with the total sperm number, the sperm concentration, the proportion of motile sperm, and the proportion of sperm with progressive motility. In other words, a high level of oxidative stress in semen (high value of sORP) was associated with low values of the total sperm number, the sperm concentration, the proportion of motile sperm, and the proportion of sperm with progressive motility. Moreover, sORP correlated positively with the proportion of tail abnormalities. In other words, a higher value of sORP predicted a high level of tail abnormalities and a low level of progressive motility necessary for ovum fertilization. Our results confirm sORP as a good marker of semen quality [16,45]. sORP is not yet a standard parameter determined in clinical practice; however, in recent years, it has been used more frequently because it can be easily determined by the MiOXYS system. sORP reflects the level of oxidative stress in the patient’s fluids and therefore can help to recognize MOSI affecting a significant portion of infertile men, including those previously diagnosed with idiopathic infertility [46].

The results of our study showed decreased platelet mitochondrial OXPHOS and reduced endogenous levels of CoQ_10_ and tocopherols, reflecting impaired mitochondrial health and the presence of oxidative stress, which may reflect impaired sperm mitochondrial function and morphology. Targeted supplementary therapy with antioxidants such as CoQ_10_ increased sperm mitochondrial ATP production, reduced oxidative stress, and improved male fertility [36,47]. Supplementation with CoQ_10_ improved sperm motility [32,36,48,49,50], reduced DNA fragmentation, and was effective in the treatment of idiopathic male infertility [33]. Resveratrol improved mitochondrial function and supported spermatogenesis [51]. Supplementation with multiple antioxidants is considered to be effective in the treatment of male infertility; however, the proper therapeutic doses remain to be determined [29]. Another perspective targeted therapy of abnormal sperm and oocyte mitochondria is mitochondrial transplantation [52]. We suppose that platelet mitochondrial bioenergetics together with CoQ_10_ concentration may be used as metabolic markers for monitoring of mitochondrial health and targeted therapy in infertile men. sORP could be a valuable clinical biomarker for diagnosing MOSI in men with reduced sperm quality.

## 4. Materials and Methods

### 4.1. Subjects

In this study, 30 men with sperm parameters below the 5th percentile according to the WHO manual 6th edition tested at a reproductive health clinic in Bratislava (GYN-FIV a.s.) were included. All men were from couples failing to achieve a pregnancy after 12 months or more of regular unprotected sexual intercourse, thereby fulfilling the criteria of infertility according to the WHO, and are denoted here as infertile. The mean age in the group of infertile men was 34.4 ± 0.9 years. The control group consisted of 16 healthy men, and the mean age was 39.2 ± 3.2 years. The inclusion criteria for infertile patients were failure to conceive during more than 12 months of unprotected intercourse, reduced sperm parameters according to the WHO 6th guideline criteria, age ≥ 25, and ≥1 million motile sperm per milliliter. The exclusion criteria were endocrine disorders, infectious diseases, genetic disorders that could affect sperm parameters, oncological diseases, chemotherapy or radiotherapy, alcohol drinking, and smoking. The criteria for the inclusion of participants into the control group were overall health (no diagnosis, no medication), blood count, and biochemical parameters in a normal range. The exclusion criteria were smoking, drinking alcohol on a regular basis, special diet, and active sport. Blood samples from all participants for the evaluation of platelet mitochondrial bioenergetics and antioxidant levels were taken in the morning after overnight fasting. On the same day, after blood sampling, semen samples were obtained from patients by masturbation. All patients were asked for 3 days of ejaculatory abstinence, and all participants had to avoid strenuous physical activity and alcohol drinking 48 h before sampling. All participants filled in a questionnaire about their health status.

### 4.2. Semen Analysis

Semen analysis was performed according to the 6th edition WHO guidelines [1]. The volume, pH of ejaculate, concentration, motility, and total sperm abnormalities were evaluated after 30 min of liquefication at 37 °C in an INCU-Line IL10 incubator (VWR, Radnor, PA, USA), as described previously [53]. The volume of the ejaculate was measured with a Pasteur pipette (Sarstedt, Nümbrecht, Germany), and the pH was determined by pH indicator paper (Ahlstrom, Espoo, Finland). All basic sperm parameters were evaluated on a Zeiss Primostar microscope (Zeiss, Jena, Germany) in a bright field of view at 200× magnification (for concentration and motility) and 1000× with immersion (for morphology). The concentration and motility were observed in the Makler chamber (Microptic, Barcelona, Spain) according to the manufacturer’s recommendations and the WHO laboratory manual for the examination and processing of human semen 6th edition [1]. Sperm count and motility were determined in 3 replicates on a minimum of 150 sperm. Abnormal morphology was evaluated on V-sperm morpho slides, i.e., ready-to-use pre-stained slides, for a distinct assessment of sperm morphology (Vitromed, Jena, Germany). In every patient, 200 sperm were evaluated for head, midpiece, and tail abnormalities. Subsequently, percentages of spermatozoa with normal morphology were evaluated according to the WHO. The static oxidation–reduction potential (sORP) of the ejaculate was evaluated by MiOXSYS^®^System [44].

### 4.3. Platelet Isolation

Blood samples were collected by venipuncture in two 9 mL K_3_EDTA (tripotassium ethylenediaminetetraacetic acid) tubes in the morning at GYN-FIV, a.s., from May to June 2022 and transported in a thermal insulation container to the Pharmacobiochemical Laboratory of the 3rd Department of Internal Medicine, Faculty of Medicine, Comenius University, Bratislava. Centrifugation of the tubes with blood was performed at room temperature at 200× *g* for 10 min using a swing-out rotor without braking. Platelet-rich plasma (PRP) was separated and subsequently transferred into a new plastic tube and mixed with 100 mM EGTA (ethylene glycol-bis(2-aminoethyl ether)-N,N,N′,N′-tetraacetic acid) to a final concentration of 10 mmol/L EGTA. The tubes were centrifuged at 1200× *g* for 10 min; then, the pellet was washed with 4 mL of DPBS (Dulbecco’s phosphate-buffered saline) plus 10 mM EGTA and centrifuged at 1200× *g* for 5 min. The pellet was resuspended in 0.4 mL of the same solution, and the platelet suspension was counted (10 times diluted) by using the hematological analyzer Mindray BC−6200 (Mindray, Shenzhen, China) [54].

### 4.4. High-Resolution Respirometry

High-resolution respirometry was used to determine the mitochondrial function in isolated platelets [55,56]. A total of 250 × 10^6^ platelets were added into a 2 mL chamber of an O2k-Respirometer (Oroboros Instruments, Innsbruck, Austria) with mitochondrial respiration medium MiR05 and 20 mM creatine. Respiration was measured at 37 °C under continuous stirring at 750 rpm [55]. Substrate–uncoupler–inhibitor titration (SUIT) reference protocol 1 was applied [17]. The titration steps were as follows: Digitonin (0.20 µg/10^6^ cells) was used for the permeabilization of the cell membrane (Dig). Mitochondrial complex I (CI)-linked LEAK respiration was measured after the subsequent addition of CI-linked substrates pyruvate (5 mM) and malate (2 mM) (1PM). CI-linked oxidative phosphorylation (OXPHOS) capacity (2D), the parameter of mitochondrial respiration associated with ATP production, was determined after the addition of saturating ADP concentration (1 mM). The addition of cytochrome c (10 μM) was used for testing the integrity of the outer mitochondrial membrane (2D;c). Maximum oxidative capacity with CI-linked substrates (CI-linked ET capacity) (3U) was evaluated after the titration of uncoupler carbonyl cyanide 4-(trifluoromethoxy)phenylhydrazone (FCCP, 0.5 μM steps). CI-linked electron transfer (ET) capacity after the addition of 10 mM glutamate (4G) and CI&CII-linked ET capacity after the addition of complex II (CII)-linked substrate succinate (10 mM) (5S) were determined. The protocol continued with the addition of an inhibitor of CI, rotenone (6Rot), allowing the determination of CII-linked ET capacity, and the addition of glycerophosphate (7Gp) for the evaluation of ET capacity associated with CII and glycerophosphate dehydrogenase (CII&GpDH). In this step, another titration of the uncoupler was required to reach the maximal ET capacity (7Gp;U). In the end, CIII inhibitor antimycin A (8Ama) was added for the evaluation of residual oxygen consumption (ROX). As the ROX after Ama was higher than the respiratory rate after digitonin (both rates representing ROX), for the evaluation of mitochondrial respiration, the respiratory rate after digitonin was subtracted from all respiratory rates.

### 4.5. Determination of Coenzyme Q_10-TOTAL_, α-tocopherol, and γ-tocopherol in Blood And Plasma

A modified HPLC method with spectrophotometric detection was used to determine the concentrations of CoQ_10-TOTAL_ (ubiquinone + ubiquinol) and tocopherols in whole blood and plasma [57,58]. First, 100 μL of 1,4-benzoquinone (2 mg/mL double-distilled water) was added to 500 μL of blood or plasma for the oxidation of ubiquinol to ubiquinone. The tubes with samples were vortexed for 10 s and incubated for 10 min at room temperature [59]; then, 2 mL of a mixture of hexane/ethanol (5:2) was added, and the tubes were shaken for 5 min. After centrifugation at 1000× *g* for 5 min, the hexane layer was separated, and the extraction process was repeated with 1 mL of the extraction mixture. Nitrogen was used to evaporate the collected organic layers at 50 °C. The residues were taken up in 99.9% ethanol and injected into a reverse-phase HPLC column (SGX C18, 7 μm, Tessek Ltd., Strašnice, Czech Republic). Ethanol/acetonitrile/ethanol (6:2:2) at a flow rate of 0.9 mL/min was used for elution. A UV detector at 275 and 295 nm, respectively, was used to detect the concentrations of CoQ_10-TOTAL_ and tocopherols. The CSW32 chromatographic station (DataApex Ltd., Prague, Czech Republic) was used to collect and process the data. The concentrations of analyzed substances were calculated in μmol/L an external standard (Sigma-Aldrich, Saint-Louis, MO, USA).

### 4.6. Determination of Coenzyme Q_10-TOTAL_, α-tocopherol, and γ-tocopherol in Platelets

Cold methanol (500 μL) was used to disintegrate the isolated human platelets (150–250 × 10^6^) [60], and 1,4-benzoquinone was added for the oxidation of ubiquinol to ubiquinone. The extraction of the cell suspension was performed with 2 mL hexane by shaking for 5 min. The organic layer was separated after centrifugation, evaporated, and measured as described above. The concentrations of analyzed substances were calculated in pmol/10^9^ cells.

### 4.7. Determination of TBARS in Plasma

A parameter of oxidative stress, thiobarbituric acid reactive substances (TBARS), was determined by the spectrophotometric method [61].

### 4.8. Statistical Analysis

The results in tables and bar graphs are expressed as mean ± standard error of the mean (sem). The graphs with correlations show individual data points. Linear correlations are expressed by the Pearson correlation coefficient *r*. The data were statistically evaluated with GraphPad Prism 6 for Windows. Normality was tested with the D’Agostino and Pearson omnibus normality test. The majority of parameters followed the normal distribution, allowing the use of an unpaired Student’s t-test for the evaluation of the difference between the infertile group and the control group. If the variables did not follow a normal distribution, the Mann–Whitney test was used. Values of *p* < 0.05 were considered statistically significant.

## 5. Conclusions

Mitochondrial dysfunction, low levels of antioxidants (CoQ_10_ and α- and γ-tocopherol), and oxidative stress are the main factors of male infertility. Platelet mitochondrial bioenergetics can reflect systemic mitochondrial health. Our pilot study showed that platelet mitochondrial bioenergetics and endogenous levels of CoQ_10_ could be used as markers of mitochondrial health in infertile men. sORP could be a valuable clinical biomarker of oxidative stress predicting the fertilization ability of human sperm.

## Figures and Tables

**Table 1:** Semen parameters, morphological defects, and a marker of oxidative stress, sORP, in the ejaculate of infertile patients. This table shows the bottom fifth percentile of data from men in couples who achieved pregnancy within one year of unprotected sexual intercourse, leading to natural conception according to WHO 6th edition, as well as the mean values ± sem in the group of infertile patients and the number (*N*) and proportion (%) of patients with values above the 5th percentile.

	Fifth Percentile According to WHO	Infertile	Values Above Fifth Percentile	
	Sixth Edition	*N* = 30	*N*	%
**Semen parameters**				
Semen volume (mL)	1.4	3.36 ± 0.23	28	93.3
pH	7.2	7.77 ± 0.05	30	100.0
Total sperm number (×10^6^)	39	93.2 ± 13.8	24	80.0
Sperm concentration (×10^6^/mL)	16	27.1 ± 3.2	23	76.7
Total motility (%)	42	56.4 ± 4.1	21	70.0
Progressive motility (%)	30	22.8 ± 2.4	7	23.3
Normal sperm morphology (%)	4.0	2.8 ± 0.3	8	26.7
**Morphological defects**				
Abnormal heads (%)	˗	92.2 ± 0.9		
Abnormal midpieces (%)	˗	43.2 ± 2.0		
Abnormal tails (%)	˗	20.4 ± 1.0		
**Oxidative status**	Normal range *		**Normal range**	
sORP (mV/10^6^ sperm/mL) ^1^	0–1.38	2.85 ± 0.95	15	50

^1^ sORP—static oxidation–reduction potential. * Normal range according to manufacturer data.

**Table 2:** Interdependence between various semen parameters in the group of infertile patients. The table shows the correlation coefficients *r* and the statistical significance (*p*-value) of the correlation between the parameters in headings.

	Sperm Concentration (10^6^/mL)	Motile Sperm Concentration (10^6^/mL)	Sperm with Progressive Motility (%)	Abnormal Sperm Morphology (%)	Head Abnormalities (%)	Midpiece Abnormalities (%)	Tail Abnormalities (%)	sORP (mV/10^6^/mL/)
Total sperm number (10^6^)	0.841	0.706	0.406	−0.326	−0.297	−0.157	−0.421	−0.594
*p*-value	<0.0001	<0.0001	0.0259	0.08	0.11	0.41	0.0204	0.0007
Sperm concentration (10^6^/mL)		0.920	0.636	−0.556	−0.510	−0.220	−0.621	−0.752
*p*-value		<0.0001	0.0002	0.0014	0.004	0.24	0.0003	<0.0001
Motile sperm concentration (10^6^/mL)			0.692	−0.658	−0.621	−0.237	−0.607	−0.637
*p*-value			<0.0001	<0.0001	0.0002	0.21	0.0004	0.0002
Sperm with progressive motility (%)				−0.406	−0.442	−0.296	−0.639	−0.582
*p*-value				0.0261	0.0144	0.11	0.0001	0.0009
Abnormal sperm morphology (%)					0.941	0.545	0.666	0.249
*p*-value					<0.0001	0.0018	<0.0001	0.19
Head abnormalities (%)						0.534	0.686	0.313
*p*-value						0.0024	<0.0001	0.10
Midpiece abnormalities (%)							0.580	0.012
*p*-value							0.0008	0.95
Tail abnormalities (%)								0.516
*p*-value								0.0042

## Data Availability

The data are contained within this article.

## References

[1] World Health Organization (2021). WHO Laboratory Manual for the Examination and Processing of Human Semen.

[2] Kumar N., Singh A.K. (2015). Trends of male factor infertility, an important cause of infertility: A review of literature. J. Hum. Reprod. Sci..

[3] Agarwal A., Baskaran S., Parekh N., Cho C.L., Henkel R., Vij S., Arafa M., Panner Selvam M.K., Shah R. (2021). Male infertility. Lancet.

[4] Mai Z., Yang D., Wang D., Zhang J., Zhou Q., Han B., Sun Z. (2024). A narrative review of mitochondrial dysfunction and male infertility. Transl. Androl. Urol..

[5] Llavanera M., Delgado-Bermudez A., Ribas-Maynou J., Salas-Huetos A., Yeste M. (2023). Reply of the Authors: A systematic review identifying fertility biomarkers in semen: A clinical approach through Omics to diagnose male infertility. Fertil. Steril..

[6] Kaltsas A., Moustakli E., Zikopoulos A., Georgiou I., Dimitriadis F., Symeonidis E.N., Markou E., Michaelidis T.M., Tien D.M.B., Giannakis I. (2023). Impact of Advanced Paternal Age on Fertility and Risks of Genetic Disorders in Offspring. Genes.

[7] Amor H., Hammadeh M.E. (2022). A Systematic Review of the Impact of Mitochondrial Variations on Male Infertility. Genes.

[8] Boguenet M., Bouet P.E., Spiers A., Reynier P., May-Panloup P. (2021). Mitochondria: Their role in spermatozoa and in male infertility. Hum. Reprod. Update.

[9] Costa J., Braga P.C., Rebelo I., Oliveira P.F., Alves M.G. (2023). Mitochondria quality control and male fertility. Biology.

[10] Chemes H.E., Alvarez Sedo C. (2012). Tales of the tail and sperm headaches: Changing concepts on the prognostic significance of sperm pathologies affecting the head, neck and tail. Asian J. Androl..

[11] Gnaiger E. (2020). Mitochondrial pathways and respiratory control. An introduction to OXPHOS analysis. 5th ed. Bioenerg. Commun..

[12] Crane F.L. (2001). Biochemical functions of coenzyme Q10. J. Am. Coll. Nutr..

[13] Mantle D., Dewsbury M., Hargreaves I.P. (2024). The Ubiquinone-Ubiquinol Redox Cycle and Its Clinical Consequences: An Overview. Int. J. Mol. Sci..

[14] Balercia G., Mancini A., Paggi F., Tiano L., Pontecorvi A., Boscaro M., Lenzi A., Littarru G.P. (2009). Coenzyme Q10 and male infertility. J. Endocrinol. Investig..

[15] Mancini A., De Marinis L., Littarru G.P., Balercia G. (2005). An update of Coenzyme Q10 implications in male infertility: Biochemical and therapeutic aspects. Biofactors.

[16] Agarwal A., Panner Selvam M.K., Arafa M., Okada H., Homa S., Killeen A., Balaban B., Saleh R., Armagan A., Roychoudhury S. (2019). Multi-center evaluation of oxidation-reduction potential by the MiOXSYS in males with abnormal semen. Asian J. Androl..

[17] Doerrier C., Garcia-Souza L.F., Krumschnabel G., Wohlfarter Y., Mészáros A.T., Gnaiger E. (2018). High-resolution FluoRespirometry and OXPHOS protocols for human cells, permeabilized fibers from small biopsies of muscle, and isolated mitochondria. Methods Mol. Biol..

[18] Gao Y., Mruk D.D., Cheng C.Y. (2015). Sertoli cells are the target of environmental toxicants in the testis—A mechanistic and therapeutic insight. Expert Opin. Ther. Targets.

[19] Tesarik J., Mendoza-Tesarik R. (2023). Mitochondria in human fertility and infertility. Int. J. Mol. Sci..

[20] Park Y.-J., Pang M.-G. (2021). Mitochondrial Functionality in Male Fertility: From Spermatogenesis to Fertilization. Antioxidants.

[21] Maharramova S., Atakishizade S., Valiyeva M., Khalilov R., Eftekhari A. (2022). Structure and function of mitochondria and its role in male infertility. Cent. Asian J. Med. Pharm. Sci. Innov..

[22] Meng K., Liu Q., Qin Y., Qin W., Zhu Z., Sun L., Jiang M., Adu-Amankwaah J., Gao F., Tan R. (2024). Mechanism of mitochondrial oxidative phosphorylation disorder in male infertility. Chin. Med. J..

[23] Bansal A.K., Bilaspuri G.S. (2010). Impacts of oxidative stress and antioxidants on semen functions. Vet. Med. Int..

[24] Koppers A.J., De Iulis G.N., Finnie J.M., McLaughlin E.A., Aitken R.J. (2008). Significance of mitochondrial reactive species in the generation of oxidative stress in spermatozoa. J. Clin. Endocrinol. Metab..

[25] Agarwal A., Parekh N., Panner Selvam M.K., Henkel R., Shah R., Homa S.T., Ramasamy R., Ko E., Tremellen K., Esteves S. (2019). Male Oxidative Stress Infertility (MOSI): Proposed Terminology and Clinical Practice Guidelines for Management of Idiopathic Male Infertility. World J. Mens Health.

[26] Shahrokhi S.Z., Salehi P., Alyasin A., Taghiyar S., Deemeh M.R. (2020). Asthenozoospermia: Cellular and molecular contributing factors and treatment strategies. Andrologia.

[27] Cedíková M., Miklíková M., Grundmanová M., Zech N.H., Králíčková M., Kuncová J. (2014). Funkce mitochondrií ve spermii u mužů s normozoospermií a astenozoospermií [Sperm mitochondrial function in men with normozoospermia and asthenozoospermia]. Ceska Gynekol..

[28] Ferramosca A., Albani D., Coppola L., Zara V. (2015). Varicocele negatively affects sperm mitochondrial respiration. Urology.

[29] Alahmar A.T., Calogero A.E., Singh R., Cannarella R., Sengupta P., Dutta S. (2021). Coenzyme Q10, oxidative stress, and male infertility: A review. Clin. Exp. Reprod. Med..

[30] Ahmadi S., Bashiri R., Ghadiri-Anari A., Nadjarzadeh A. (2016). Antioxidant supplements and semen parameters: An evidence based review. Int. J. Reprod. Biomed..

[31] Salvio G., Cutini M., Ciarloni A., Giovannini L., Perrone M., Balercia G. (2021). Coenzyme Q10 and male infertility: A systematic review. Antioxidants.

[32] Gvozdjáková A., Dúbravický J., Singh R.B., Gvozdjáková A., Cornélissen G., Singh R.B. (2018). Mitochondrial reproductive medicine. Recent Advances in Mitochondrial Medicine and Coenzyme Q10.

[33] Alahmar A.T., Calogero A.E., Sengupta P., Dutta S. (2021). Coenzyme Q10 improves sperm parameters, oxidative stress markers and sperm DNA fragmentation in infertile patients with idiopathic oligoasthenozoospermia. World J. Mens Health.

[34] Alahmar A.T., Sengupta P. (2021). Impact of coenzyme Q10 and selenium on seminal fluid parameters and antioxidant status in men with idiopathic infertility. Biol. Trace Elem. Res..

[35] Kobori Y., Ota S., Sato R., Yagi H., Soh S., Arai G., Okada H. (2014). Antioxidant cosupplementation therapy with vitamin C, vitamin E, and coenzyme Q10 in patients with oligoasthenozoospermia. Arch. Ital. Urol. Androl..

[36] Gvozdjáková A., Kucharská J., Dubravicky J., Mojto V., Singh R.B. (2015). Coenzyme Q₁₀, α-tocopherol, and oxidative stress could be important metabolic biomarkers of male infertility. Dis. Markers.

[37] Zhou X., Shi H., Zhu S., Wang H., Sun S. (2022). Effects of vitamin E and vitamin C on male infertility: A meta-analysis. Int. Urol. Nephrol..

[38] Marin-Guzman J., Mahan D.C., Pate J.L. (2000). Effect of dietary selenium and vitamin E on spermatogenic development in boars. J. Anim. Sci..

[39] Sabetian S., Jahromi B.N., Vakili S., Forouhari S., Alipour S. (2021). The effect of oral vitamin E on semen parameters and IVF outcome: A double-blinded randomized placebo-controlled clinical trial. Biomed. Res. Int..

[40] Traber M., Pauling A.H. (2015). “Vitamin E”. https://lpi.oregonstate.edu/mic/vitamins/vitamin-E.

[41] Jiang Q., Im S., Wagner J.G., Hernandez M.L., Peden D.B. (2022). Gamma-tocopherol, a major form of vitamin E in diets: Insights into antioxidant and anti-inflammatory effects, mechanisms, and roles in disease management. Free Radic. Biol. Med..

[42] Schmid T.E., Eskenazi B., Marchetti F., Young S., Weldon R.H., Baumgartner A., Anderson D., Wyrobek A.J. (2012). Micronutrients intake is associated with improved sperm DNA quality in older men. Fertil. Steril..

[43] Keshtgar S., Fanaei H., Bahmanpour S., Azad F., Ghannadi A., Kazeroni M. (2012). In vitro effects of α-tocopherol on teratozoospermic semen samples. Andrologia.

[44] Agarwal A., Sharma R., Roychoudhury S., Du Plessis S., Sabanegh E. (2016). MiOXSYS: A novel method of measuring oxidation reduction potential in semen and seminal plasma. Fertil. Steril..

[45] Agarwal A., Roychoudhury S., Sharma R., Gupta S., Majzoub A., Sabanegh E. (2017). Diagnostic application of oxidation-reduction potential assay for measurement of oxidative stress: Clinical utility in male factor infertility. Reprod. Biomed. Online.

[46] Takalani N.B., Monageng E.M., Mohlala K., Monsees T.K., Henkel R., Opuwari C.S. (2023). Role of oxidative stress in male infertility. Reprod. Fertil..

[47] Gvozdjáková A., Kucharská J., Lipková J., Bartolčičová B., Dubravický J., Voráková M., Černáková I., Singh R.B. (2013). Importance of the assessment of coenzyme Q10, alpha-tocopherol and oxidative stress for the diagnosis and therapy of infertility in men. Bratisl. Med J..

[48] Gvozdjáková A., Gvozdjáková A. (2008). Mitochondrial “Spermatopathy”. Mitochondrial Medicine.

[49] Vishvkarma R., Alahmar A.T., Gupta G., Rajender S. (2020). Coenzyme Q10 effect on semen parameters: Profound or meagre?. Andrologia.

[50] Alharbi M. (2024). Impact of Antioxidants on Conventional and Advanced Sperm Function Parameters: An Updated Review. Cureus.

[51] Illiano E., Trama F., Zucchi A., Iannitti R.G., Fioretti B., Costantini E. (2020). Resveratrol-based multivitamin supplement increases sperm concentration and motility in idiopathic male infertility: A pilot clinical study. J. Clin. Med..

[52] Morimoto Y., Gamage U.S.K., Yamochi T., Saeki N., Morimoto N., Yamanaka M., Koike A., Miyamoto Y., Tanaka K., Fukuda A. (2023). Mitochondrial transfer into human oocytes improved embryo quality and clinical outcomes in recurrent pregnancy failure cases. Int. J. Mol. Sci..

[53] Kubikova E., Klein M., Svitok P., Stefanic J., Benus R., Varga I. (2019). Fertility maintenance in male oncological patients: Current state and future perspectives. Bratisl. Med. J..

[54] Sumbalova Z., Kucharska J., Palacka P., Rausova Z., Langsjoen P.H., Langsjoen A.M., Gvozdjakova A. (2022). Platelet mitochondrial function and endogenous coenzyme Q10 levels are reduced in patients after COVID-19. Bratisl. Med. J..

[55] Pesta D., Gnaiger E. (2012). High-resolution respirometry: OXPHOS protocols for human cells and permeabilized fibers from small biopsies of human muscle. Methods Mol. Biol..

[56] Sjövall F., Ehinger J.K., Marelsson S.E., Morota S., Frostner E.A., Uchino H., Lundgren J., Arnbjörnsson E., Hansson M.J., Fellman V. (2013). Mitochondrial respiration in human viable platelets--methodology and influence of gender, age and storage. Mitochondrion.

[57] Lang J.K., Gohil K., Packer L. (1986). Simultaneous determination of tocopherols, ubiquinols, and ubiquinones in blood, plasma, tissue homogenates, and subcellular fractions. Anal. Biochem..

[58] Kucharská J., Gvozdjáková A., Mizera S., Braunová Z., Schreinerová Z., Schrameková E., Pecháň I., Fabián J. (1998). Participation of coenzyme Q10 in the rejection development of the transplanted heart. Physiol. Res..

[59] Mosca F., Fattorini D., Bompadre S., Littarru G.P. (2002). Assay of coenzyme Q10 in plasma by a single dilution step. Anal. Biochem..

[60] Niklowitz P., Menke T., Andler W., Okun J.G. (2021). Simultaneous analysis of coenzyme Q10 in plasma, erythrocytes and platelets: Comparison of the antioxidant level in blood cells and their enviroment in healthy children and after oral supplementation in adults. Clin. Chim. Acta.

[61] Janero D.R., Bughardt B. (1989). Thiobarbituric acid-reactive malondialdehyd formation during suproxide-dependent, iron-catalyzed lipid peroxidation: Influence of peroxidation conditions. Lipids.

